# Clinician Experiences With Ambient Scribe Technology to Assist With Documentation Burden and Efficiency

**DOI:** 10.1001/jamanetworkopen.2024.60637

**Published:** 2025-02-19

**Authors:** Matthew J. Duggan, Julietta Gervase, Anna Schoenbaum, William Hanson, John T. Howell, Michael Sheinberg, Kevin B. Johnson

**Affiliations:** 1 Department of Information Services, University of Pennsylvania Health System, Philadelphia; 2 Sidney Kimmel Medical College, Thomas Jefferson University, Philadelphia, Pennsylvania; 3 Hospital of the University of Pennsylvania, Philadelphia; 4 Lancaster General Health, University of Pennsylvania, Philadelphia; 5 Division of Biomedical Informatics, Department of Biostatistics, Epidemiology, and Informatics, Perelman School of Medicine, University of Pennsylvania, Philadelphia; 6 Department of Pediatrics, Children’s Hospital of Philadelphia, Philadelphia, Pennsylvania

## Abstract

**Importance:**

Timely evaluation of ambient scribing technology is warranted to assess whether this technology can lessen the burden of clinical documentation on clinicians.

**Objective:**

To investigate the association of ambient scribing technology with efficiency, quality, and perceived burden of clinical documentation in the outpatient setting.

**Design, Setting, and Participants:**

This prospective, single-group pre-post quality improvement study was conducted between April and June 2024 in the outpatient setting of an academic health system in Philadelphia, Pennsylvania. Participants included physicians, nurse practitioners, and physician assistants. Data were analyzed from July to August 2024.

**Exposure:**

Access to an artificial intelligence–driven ambient scribing tool for outpatient notes.

**Main Outcomes and Measures:**

The primary outcomes were time in notes per appointment, same-day appointment closure, after-hours work time, perceived burden of clinical documentation, and comments on clinicians’ experiences using ambient scribing. A mixed-effects model was used. Both objective metrics and survey feedback were obtained. Targeted perspective questions, designed to assess clinician-perceived patient engagement and perceived documentation burden, were assessed on a 7-point Likert scale. Standard System Usability Scale (SUS) and net promoter score (NPS) formulas were used to analyze usability and recommendability data.

**Results:**

This study included 46 clinicians from 17 different medical specialties, with a mean (SD) of 11.1 (8.7) years in practice. From baseline to post intervention, use of the ambient scribing tool was associated with 20.4% less time in notes per appointment (from 10.3 to 8.2 minutes; *P* < .001), 9.3% greater same-day appointment closure (from 66.2% to 72.4%; *P* <.001), and 30.0% less after-hours work time per workday (from 50.6 to 35.4 minutes per workday; *P* = .02). Targeted perspective questions showed more favorable scores. Open-ended qualitative feedback from clinicians showed a range of positive, negative, and mixed feedback regarding their use of ambient scribing technology. SUS scores showed that the ambient scribing tool was easy to use. NPSs reflected the mixed qualitative feedback.

**Conclusions and Relevance:**

In this quality improvement study, the use of ambient scribe technology was associated with greater efficiency of outpatient clinical documentation, lower mental burden of documentation for clinicians, and greater sense of engagement with patients during outpatient appointments. Additional studies exploring urgent care settings, examining patient experience, and comparing multiple tools will be important to better understand the effect of ambient scribing on ambulatory care.

## Key Points

**Question:**

What is the association of ambient scribe technology with scribing efficiency and clinical documentation burden for clinicians in the outpatient setting?

**Findings:**

In this quality improvement study with 46 participants, use of an ambient scribe tool was associated with greater clinician efficiency, lower mental burden of documentation, and greater sense of engagement with patients during outpatient appointments.

**Meaning:**

These findings suggest that ambient scribing has the potential to measurably decrease the burden of clinical documentation, which is a substantial source of clinician burnout.

## Teaser

This quality improvement study assesses clinician experiences with ambient scribing technology and its association with efficiency and clinical documentation burden in the outpatient setting.

## Introduction

Clinical documentation in electronic health records (EHRs) is a substantial source of clinician burnout.^1,2^ Prior studies have shown that this burnout is driven by a number of factors related to clinical documentation, including the amount of time and effort required during and after visits,^3,4^ its interference with patient interaction during appointments,^2^ and the usability of EHR software in which documentation occurs.^1,4,5^ A key strategy to address this challenge is the use of team-based documentation, such as medical scribes.^6,7^ Recently published work^8^ suggests that ambient scribe tools also may help reduce this burden. Ambient scribe tools “listen” to a clinical encounter between a clinician and a patient and use generative artificial intelligence (AI) to summarize the encounter with immediate note delivery. Some recent studies show favorable feedback from clinicians^9^ and improvements in simulated appointments,^10^ whereas other studies show more neutral outcomes.^11,12^ To date, no existing studies have integrated robust usage data with high response rate qualitative feedback about clinicians’ experiences using the most current versions of this technology. The University of Pennsylvania is among the first health care systems to collaborate with an EHR vendor to pilot the use of an EHR-integrated ambient scribe tool in outpatient clinical practice. The goal of this study was to quantitatively and qualitatively assess clinician experiences using ambient scribing as a clinical documentation assistant in an outpatient clinical setting.

## Methods

This pre-post quality improvement study was conducted at an academic medical center and was deemed exempt from further review following its classification as a quality improvement initiative by the University of Pennsylvania Institutional Review Board. Ambient scribe technology was offered to a group of clinicians for use during outpatient medical appointments. Answering a baseline preuse survey served as implied consent from health care professionals to participate in the project. Patients verbally consented to clinicians’ use of the ambient scribing tool during clinical encounters; however, at no point during the study was any patient information collected or reviewed by the project team. This study followed the Standards for Quality Improvement Reporting Excellence (SQUIRE) reporting guideline.

### Recruitment and Enrollment of Study Participants

Potential participants were identified through a combination of informal recruiting emails to identify interested clinicians as well as nominations from department leaders. Project team members evaluated interested clinicians to ensure a mix of medical specialties (17 unique specialties), clinical sites (22 unique sites), and clinician scribing skill levels. Scribing skill level was determined using data from an analytics tool (Signal; Epic Systems) that automatically tracks how clinicians use the Epic EHR system (Epic Systems). Recruitment identified 46 clinicians, all of whom enrolled in the 7-week pilot program, consisting of a 2-week implementation and clinician training period followed by 5 weeks of ambient scribe usage. During the training period, all participants attended a 30- to 60-minute small group orientation via video call, during which dedicated educators taught them how to use the ambient scribe tool. Participating clinicians used ambient scribing for primary care and specialist outpatient appointments, with ongoing technological support available from support staff.

### Integration of Ambient Scribing Into the Patient Visit

Clinicians were given access to a commercially available ambient scribe tool (DAX Copilot; Nuance), integrated directly with the EHR. During this pilot, clinicians used an EHR system mobile application (Haiku; Epic Systems) on a mobile device to maintain consistent and flexible access to a microphone for audio recording. Clinicians with ambient scribe access were able to activate a recording button within the EHR mobile application patient-visit interface to begin recording. Clinicians confirmed consent to audio recording with the patient and any other parties present in the encounter before starting passive, hands-free recording of the visit. At the end of the visit, the clinician stopped the recording, after which AI automatically interpreted the conversation and produced a full template or partial note section (eg, history of present illness, physical examination, assessment, and plan) to be available to the clinician to review most often in less than 1 minute.

### Outcomes Assessment

The EHR analytics tool was used to obtain participant EHR usage patterns from the 5-week study period and from a 9-month baseline period prior to the initiation of the study. The analytics tool was used to obtain EHR efficiency metrics for each participant weekly, covering 9 months before beginning to use ambient scribing (to generate a baseline for each clinician) and for 5 weeks during the study (to assess participant behavior while using ambient scribing). The efficiency metrics were as follows: clinician average time in notes per appointment, percentage of appointments closed the same day, and average after-hours work time per workday (also known as “pajama time”). In addition, the analytics data included the percentage of documentation characters entered by the clinician, which measured the proportion of the note manually typed by the clinician relative to the total characters in all the clinician’s notes during a given week (excludes spaces, tabs, copy-pasted characters, and dictation). The analytics data also included a measurement of overall note length, as represented by total characters in all notes generated by the clinician during a given week (excludes spaces and tabs; includes copy-pasted characters, dictation, manually typed characters, and ambient scribing–generated characters). Finally, the analytics data were also used to measure EHR system and ambient scribe usage over the course of the study (eFigure in Supplement 1).

Surveys were administered before and after the intervention to participants who used ambient scribing during this period. The preuse and postuse surveys contained a set of targeted perspective questions (TPQs) generated by the project team to elucidate aspects of the clinicians’ experiences that were not otherwise available through EHR metrics. Responses to these questions were measured on a 7-point Likert scale. Changes in the average score are reported, along with the number of clinicians who changed their opinion (switching from an agree to a disagree score, or vice versa) between the preuse and postuse surveys. Postuse surveys also included open-ended qualitative feedback, validated survey instruments measuring usability with the Systems Usability Scale (SUS),^13^ and likelihood to recommend ambient scribing to their colleagues, the latter of which was used to calculate a net promoter score (NPS).^14^

### Qualitative Analysis

Thematic analysis was performed on the postuse survey comments to identify factors associated with the results of the TPQs, SUS, NPS, and EHR analytics data. Researchers and ChatGPT, version 4.0 (OpenAI), analyzed this written feedback from July 25 to July 27, 2024 (the ChatGPT text prompt is provided in the eMethods in Supplement 1). Consensus on themes was reached by relating comments back to the relevant component of the other analyses for validation.

### Statistical Analysis

Random-effects modeling was used to analyze the EHR analytics data using Python, version 3.10 (Python Software Foundation) (with the SciPy, version 1.11.4, and statsmodels, version 0.13.2, packages). Covariates for this analysis included clinician role, medical specialty, patient complexity (number of active problems on patient problem list), clinician workload (average number of appointments per week), and years of clinician experience. Our primary random-effects model excluded the number of weeks post intervention when clinicians had no recorded use of the ambient scribe tool; results from 2 additional models are included in the eTable in Supplement 1 for sensitivity analyses where no exclusions were made (model A) and a shorter baseline period was used (model B).

For the Likert-scored TPQs, 2-sided *t* tests were performed using Microsoft Excel (Microsoft Corp), and average scores were compared before and after ambient scribing usage. Statistical significance was defined as *P* < .05 (2-tailed). The number of respondents who changed from a score indicating agreement or neutrality to disagreement (or vice versa) is also reported. Standard SUS and NPS formulas were used to analyze the usability and recommendability data.

## Results

Preuse survey responses were used as a prerequisite to enrollment, resulting in a response rate of 100% (46 of 46 pilot clinicians). The postuse survey response rate was 80.4% (37 of 46 pilot clinicians). Additionally, EHR analytics data for all participating clinicians were collected before and during their use of ambient scribing, and they showed consistent usage of ambient scribing from week to week over the entire study period (eFigure in Supplement 1). The 46 participants in this study came from 17 different medical specialties. They had a mean (SD) of 11.1 (8.7) years in practice and saw a mean (SD) of 36.5 (18.2) patients per week; patients had a mean (SD) of 10.0 (4.1) active problems in the EHR (Table 1).

**Table 1.** Participant Demographics

Characteristic	Value^a^
Clinical work, mean (SD)	
Time in practice, y	11.1 (8.7)
Workload, No. of visits/wk	36.5 (18.2)
Patient complexity, No. of problems on problem list	10.0 (4.1)
Clinician role	
Physician	40 (87.0)
Nurse practitioner	2 (4.3)
Physician assistant	4 (8.7)
Specialty (self-reported)^b^	
Allergy and immunology	1 (2.2)
Dermatology	1 (2.2)
Endocrinology	2 (4.3)
Family medicine	17 (37.0)
Gastroenterology	1 (2.2)
Geriatric medicine	2 (4.3)
Hematology and oncology	3 (6.5)
Nephrology	3 (6.5)
Neurosurgery	1 (2.2)
Obstetrics and gynecology	1 (2.2)
Ophthalmology	2 (4.3)
Orthopedic surgery	6 (13.0)
Pediatric medicine	1 (2.2)
Pulmonary disease or pulmonology	2 (4.3)
Rheumatology	1 (2.2)
Urgent care specialist	2 (4.3)
Urology	2 (4.3)

^a^ Unless indicated otherwise, values are presented as No. (%) of clinicians.

^b^ Values sum to more than 100% because participants could indicate more than 1 specialty.

On the standardized SUS, clinicians gave ambient scribing a mean (SD) rating of 76.6 (16.1) of 100, indicating that they found ambient scribing easy to use. When asked how likely they were to recommend ambient scribing to others (scale of 0-10 to quantify an NPS), 13 clinicians (35.1%) were promoters (score of 9 or 10), 11 (29.7%) were passives (score of 7 or 8), and 13 (35.1%) were detractors (score of ≤6); this equated to an overall NPS of 0 on a scale of −100 to 100.

Table 2 summarizes the outcomes associated with ambient scribing. The use of the ambient scribing tool was associated with 20.4% less time working on notes per appointment compared with baseline (from 10.3 to 8.2 minutes; *P* < .001). The same-day appointment closure rate was 9.3% greater with ambient scribing use compared with baseline (from 66.2% to 72.4%; *P* < .001). Weekly after-hours work time per workday was 30.0% less with the use of ambient scribing compared with baseline (from 50.6 to 35.4 minutes per workday; *P* = .02). Note length was 20.6% greater with ambient scribing use compared with baseline (from 202 637.5 to 244 427.1 characters per week; *P* < .001); however, the percentage of clinical documentation that was typed by clinicians was 29.6% lower compared with baseline (from 11.2% to 7.9%; *P* < .001) (Table 2). Associations between ambient scribing and all metrics persisted after controlling for clinician specialty, patient complexity, clinician workload, and clinician experience. Two alternate models (eTable in Supplement 1) yielded similar results for most outcomes.

**Table 2.** Results of Random-Effects Model for Outcomes Before vs After Intervention^a^

Outcome	Ambient scribing intervention period		Estimate, β (SE) [95% CI]	Change, %	*P* value
	Before	After			
Time in notes per appointment, min, mean (SD)	10.3 (9.2)	8.2 (6.8)	−1.6 (0.4) [−2.3 to −0.8]	−15.2	<.001
Appointments closed same day, mean (SD), %	66.2 (31.0)	72.4 (29.4)	6.0 (1.6) [2.9 to 9.2]	9.1	<.001
After-hours work time per scheduled day (pajama time), mean (SD), min	50.6 (64.3)	35.4 (52.0)	−8.6 (3.6) [−15.7 to −1.4]	−16.9	.02
Portion of notes manually typed, mean (SD), %	11.2 (9.7)	7.9 (6.5)	−2.3 (0.4) [−3.1 to −1.5]	−20.7	<.001
Total length of all notes generated by clinician, mean (SD), No. of characters/wk	202 637.5 (186 947.7)	244 427.1 (199 403.6)	31 390.0 (5379.3) [20 846.7 to 41 933.2]	15.5	<.001

^a^ Covariates for all outcomes included individual clinician, clinician role, specialty, patient complexity, clinician workload, and clinician experience.

On a 7-point Likert scale, the use of ambient scribing technology was associated with lower sense of distractedness due to documentation during patient conversations. Clinicians were also less likely to report feeling mentally overloaded and “drained” by the burden of clinical documentation. Clinicians were more likely to report feeling able to document patient conversations quickly, efficiently, and with the level of detail they would like when using ambient scribing technology. There was no clear association between the intervention and clinicians’ reported ability to increase patient volume or their perception of AI’s ability to record and interpret a conversation appropriately (Table 3).

**Table 3.** Targeted Perspective Questions Before and After Ambient Listening

Question	Ambient listening rating, average		Change (SE)	*P* value	Opinion change, No. of participants	
	Before	After			New agrees	New disagrees
Ambient listening could make a meaningful and positive impact on my workflow.	6.02	6.08	0.06 (0.26)	.10	2	3
I am confident that artificial intelligence can record and interpret a conversation appropriately.	4.78	4.86	0.08 (0.30)	.85	6	5
Artificial intelligence technology like ambient listening is currently ready to deploy into practice.	4.74	5.00	0.26 (0.34)	.62	10	6
I foresee ambient listening as a significant solution to provider charting burden.	5.76	5.89	0.13 (0.26)	.84	3	2
I feel distracted by documentation while I am talking with patients.	5.67	2.27	−3.40 (−0.30)	<.001	1	30
Documentation prevents me from being fully engaged and present in my conversations with patients.	5.41	2.05	−3.36 (−0.31)	<.001	1	31
I feel mentally overloaded by interacting with patients in addition to keeping up with documentation.	5.72	2.32	−3.39 (−0.32)	<.001	1	31
Documentation needs impact my ability to talk face-to-face with my patients.	5.61	2.19	−3.42 (−0.28)	<.001	0	30
My documentation burden prevents me from achieving a better work-life balance.	6.48	2.84	−3.64 (−0.32)	<.001	0	27
Documenting my encounters feels generally draining.	6.17	3.08	−3.09 (−0.32)	<.001	0	25
Completing the subjective or HPI is a major reason why I do not close visit encounters sooner.	4.52	3.08	−1.44 (−0.42)	<.001	1	15
I capture a comprehensive and thorough subjective or HPI in my notes.	4.76	5.19	0.43 (0.35)	.29	8	4
I am able to document a conversation with a patient quickly and efficiently.	3.63	5.03	1.40 (0.35)	<.001	17	3
I feel able to record as much detail in my documentation as I would like.	3.28	4.84	1.56 (0.39)	<.001	21	5
I feel able to keep up with my current patient volume.	4.07	4.89	0.83 (0.36)	.003	7	2
I feel as though I could reasonably see more patients on a scheduled day.	3.26	3.11	−0.15 (−0.39)	.93	6	8

Abbreviation: HPI, history of present illness.

### Efficiency and Time Savings

In their qualitative comments, clinicians reported greater efficiency in their workflow when using ambient scribing as well as reduced time spent on note-writing and reduced visit time (Table 4). This aligns with the findings from the EHR analytics tool showing that ambient scribing was associated with less time in notes and greater same-day appointment closure rates (Table 2), as well as TPQ analysis showing greater perceived efficiency and lower likelihood to report that unfinished documentation stopped clinicians from closing visit encounters (Table 3).

**Table 4.** Key Themes From Qualitative Feedback

Theme	Tone^a^	Representative physician comments
Efficiency and time savings due to ambient scribe tool (DAX Copilot)	+	“I am efficient to begin with, but this is saving me 15 min a day.”
	+	“It does save me a little bit of time on charting… perhaps cutting back on my documentation time by about 2 hours cumulative, each week.”
	+/−	“As a note-taking entity-program, I think it’s very valuable and I will continue to use it. However, it simply cannot create a final note product appropriate for closure and billing.”
	–	“Far less time-saving than I had anticipated.”
Patient engagement	+	“It has dramatically decreased my documentation burden and allowed me to have conversations with patients that don’t require me to divert attention from the computer screen.”
	+	“AI documentation markedly facilitates better patient interaction and efficiency.”
	–	“I did not find the physical reporting helpful as I sometimes feel awkward stating physical exam findings during my exam.”
Documentation burden	+	“When using DAX, I feel like I am more motivated to close the note right away because it does not feel like such a heavy lift (compared to doing it all on my own).”
	+	“DAX has made such a positive impact on my practice and ability to keep up with the workload.”
	+/−	“I do find I need to review and manipulate the DAX-generated notes after the visit… still, I am happy to be able to tweak notes instead of generating them from scratch at the end of a long session/day.”
	+/−	“It’s not perfect, but it is a great start. It has greatly reduced the amount of work I bring home with me.”
	+/−	“Although the amount of time saved might not be that much, [DAX plus my editing] is a better note… I also think it allows me to not rely on my memory, which relieves significant mental burden.”
	–	“The amount of time spent checking and correcting the generated text is equal to or exceeds the charting burden experienced without DAX, and has greater potential for charting errors.”
Quality of generated notes	+	“I did not have to type any history. I quickly became comfortable that it would capture all critical elements of the conversation.”
	+	“The subjective portion is my most used feature of DAX. The subjective portion was correct 80%-85% of the time.”
	–	“It tries to paraphrase the conversation, and often does it in a way that utilizes layman’s terms rather than medical terms; and often incorrectly documents what was discussed. This means that I must edit the content substantially because it cannot be used as-is in my closed note.”
	–	“The way DAX transcribes the HPI is still quite wordy and unnatural in my opinion… I find that I have to spend a good deal of time editing what DAX writes in order to suit my style.”
	–	“The language is still odd, too formal/professorial. Unfortunately, the HPI can be quite inaccurate at times and it is necessary to review it almost immediately to make corrections while it is still fresh in memory.”
	–	“It is quite inconsistent, some long conversations are whittled down to 1 or 2 short sentences. I do not think it is enough documentation for medico-legal purposes.”
	–	“Unfortunately, the output required a lot of modification (re-organization if HPI/ROS, deleting redundant and extraneous information, changing some language that is not generally utilized in medicine, and placing the results in the right section).”
Usability of ambient scribe tool (DAX Copilot), now and in the future	+	“I legitimately think this technology, once optimized, is the biggest advancement for outpatient primary care providers in decades.”
	+	“I am confident that future updates will be even more helpful.”
	+	“I anticipate [AI documentation] improvements which will improve the output and decrease proofreading burden as the technology continues to improve.”
	+/−	“Used with appropriate expectations, it is useful today. It is not yet a ‘keyboard-free’ experience, nor a significant time saver.”
	–	“I feel that AI can achieve the goals set for here, and that there are softwares that are ready to be deployed for this purpose, but DAX is not one of them. Would not recommend for use system-wide.”
	–	“I feel like for OB it won’t be as useful—these are templated visits, and the biggest charting burden for these is the problem lists, which this AI doesn’t solve.”

Abbreviations: AI, artificial intelligence; HPI, history of present illness; OB, obstetrics; ROS, review of systems.

^a^ Plus signs indicate positive tone; minus signs, negative tone; and plus/minus signs, neutral tone.

### Patient Engagement

Clinicians felt that using ambient scribing allowed them to engage more with patients without the distraction of typing (Table 4). This aligned with TPQ findings that reflected the clinicians’ sense of greater engagement and less distraction from documentation during patient interactions with ambient scribing use (Table 3).

### Documentation Burden

In their open-ended feedback, clinicians explained that their ambient scribing–generated notes did not eliminate the burden of clinical documentation. However, they did share that ambient scribing decreased the mental effort required for their documentation, both by recording difficult-to-remember details and by eliminating the difficult task of writing a note completely from scratch (Table 4). These comments align with the quantitative results in Table 2, in which ambient scribing was associated with less time in notes, less after-hours documentation time, and a lower percentage of characters typed by the clinicians themselves. Similarly, TPQ responses showed that clinicians were less likely to feel mentally overloaded by the demands of documentation and were less likely to view documentation as a barrier to work-life balance with the use of ambient scribing (Table 3).

### Quality of Generated Notes

Overall, clinicians gave mixed feedback regarding the length and quality of ambient scribe-generated notes. Some clinicians found them accurate and detailed, whereas others found them overly error-prone. A recurring theme was the need for substantial editing and proofreading of the AI-generated notes, which sometimes offset the time saved (Table 4). Despite the varied qualitative feedback on the quality of the generated notes, responses to the TPQs showed that ambient scribing was associated with greater likelihood that clinicians could record their desired amount of detail in their documentation (Table 3).

### Usability of Ambient Scribing

Clinicians gave mixed feedback about the ambient scribing technology. For example, one clinician hailed it as “the biggest advancement for outpatient primary care providers in decades,” whereas others reported feeling that “it won’t be as useful” for their medical specialty. Although some comments addressed ambient scribing technology in general, others described limitations they believed were specific to the ambient scribe tool and not necessarily universal to all ambient scribing tools (Table 4). This feedback aligned with our calculated NPS of 0. Qualitative analysis focused mostly on the usability of the generated notes rather than on the usability of the ambient scribe application itself; however, on a separate postuse survey question, the ambient scribe tool scored a mean (SD) of 76.6 (16.1) of 100 on the standardized SUS, as noted earlier.

## Discussion

Overall, in this study, ambient scribing was associated with greater efficiency, lower perceived mental burden of documentation, and greater clinician sense of engagement with patients. Other ambient scribing studies have shown a variety of outcomes, including favorable postuse survey feedback from clinicians,^10^ improved documentation and patient interaction in simulated appointments,^11^ and mixed to minimal changes from ambient scribing use.^12,13^ Our study combined both objective metrics and survey feedback from the same group of clinicians to paint a more comprehensive picture of health care clinicians’ experiences using ambient scribing technology. Our findings suggest that ambient scribing is helpful in several important ways. According to the EHR metrics, ambient scribing boosted note-writing efficiency; TPQ responses also suggested that this technology was associated with reduced cognitive burden of documentation on clinicians. However, the postuse survey feedback results also indicated that ambient scribing was not yet providing a keyboardless experience. To some clinicians, the need to review and edit was insignificant, but for others, it was cited as a reason not to use the current version of ambient scribing.

Clinicians reported using the notes generated by ambient scribing to help recall their patient encounters with a level of detail they would not have captured themselves. Similarly, many clinicians noted that it was much easier to build from ambient scribing–generated notes rather than write a new note entirely from scratch. Although these functions of the ambient scribing–generated notes were viewed positively by clinicians, it is important to acknowledge potential downsides of these findings. For example, our results suggest that the time and mental effort saved with ambient scribing was associated with a better clinician experience and increased focus on patients; however, it is also possible that the use of ambient scribing–generated notes could decrease clinicians’ opportunities to reflect while note-writing and address missed diagnoses or treatments. Additionally, the association observed between ambient scribing and longer note length may raise concern for “note bloat,” or the inclusion of unnecessary or repetitive details that obscure the most medically important information. However, increased note length^15^ and note bloat have also been observed with the use of medical scribes, so this is not an issue unique to notes generated by ambient scribing AI.^16^

Notably, the clinicians participating in this study did exhibit optimistic prepilot opinions about ambient scribing AI tools, which may have biased the results. Despite their initial optimism, survey data detected lower optimism among some clinicians after using ambient scribing; these clinicians may have come from specialties whose workflow was less conducive to the current ambient scribing (nontemplated) approach or may have had unrealistic expectations about the tool before access was granted. For this pilot study, training sessions were focused on usage and were brief because ambient scribing was relatively easy for clinicians to use; however, for larger future rollouts, adding education that highlights ambient scribing’s current capabilities and shortcomings may decrease clinician disappointment or frustration with certain aspects of these tools. Notably, there are many near-term advances planned for ambient scribing technologies; this makes the external validity of a larger trial time dependent until there is a degree of stability in what is generally available. We recommend creating a pulse survey to provide periodic updates to the community as this technology matures, as was done recently for work assessing changes in physician burnout.^17^

Our results suggest that current ambient scribing products may be a better fit for some clinicians or organizations than others. Clinicians who are seeking a fully keyboardless solution, who are particular about the style or tone of their notes, or whose specialty uses hyperspecific checklist templates for notes (eg, prenatal care checklist, Medicare Wellness Visit checklist) may find current ambient scribing tools unsatisfying. Although ambient scribing tools will likely improve and gain more features over time, in the current state, users appropriately expressed a need to proofread and edit their notes. Thus, we strongly recommend that unless the licensing and training costs change, new health care system adopters should invest in these technologies strategically, perhaps by taking into account individual-based factors that may affect a clinician’s likelihood of adopting and benefitting from the use of ambient scribing technology over other team-based or template-based documentation approaches.

### Limitations

This study has limitations. It was performed within 1 health system in the same geographic area and had a short duration, which could affect the type and variety of case patients seen by physicians and the value of ambient scribing. Also, our participants all opted in to participate. Although we controlled for a variety of covariates, bias introduced by participants’ interest in the study may mean they are not representative of the wider clinician population.

Additionally, outcome selection in this study was largely based on clinician perceptions, potential areas of clinician-level impact anticipated by the research team, and data availability via the EHR analytics tool. Future work would benefit from the inclusion of structural, process, and patient outcomes. It would also benefit from observing a larger sample size of physicians over a longer study period using a stable version of ambient scribing.

Also, there were weeks when clinicians performed outpatient charting in the EHR system but did not use ambient scribing (eFigure in Supplement 1). We were unable to confirm why: they may have opted out of using ambient scribing that week, been assigned to a service where ambient scribing use was inappropriate (eg, inpatient service), or some other scenario.

Most importantly, this study uses clinicians’ perception of patient interactions rather than directly measuring patients’ experiences. To ethically scale ambient scribing tools in a clinical setting, it will be critical to evaluate their effects on patient experience and outcomes.

## Conclusions

In this quality improvement study, use of an ambient scribe tool to draft outpatient visit summaries was associated with greater clinician efficiency, lower perceived mental burden of documentation, and greater sense of engagement with patients. Clinicians expressed differing opinions about the accuracy and completeness of the notes; however, on average, these clinicians viewed ambient scribing positively after using it for 7 weeks. These findings are encouraging and provide support for longitudinal trials across a variety of subspecialties to fully assess the effectiveness of ambient scribing across a health system, which will be important to justify costs in the current business models for purchasing this technology. Ongoing feedback from clinicians and patients is needed to inform future development and strategic investments in ambient scribing.

## Notes

Supplement 1. **eFigure.** Number of Clinicians Using Ambient Scribing Week to Week Over the Course of the Study **eTable.** Signal Data Analysis with Alternate Random-Effects Models
eMethods

Supplement 2.
Data Sharing Statement

## References

[1] Gaffney A, Woolhandler S, Cai C, . Medical documentation burden among US office-based physicians in 2019: a national study. JAMA Intern Med. 2022;182(5):564-566. doi:10.1001/jamainternmed.2022.0372 35344006 PMC8961402

[2] Budd J. Burnout related to electronic health record use in primary care. J Prim Care Community Health. 2023;14:21501319231166921. doi:10.1177/21501319231166921 37073905 PMC10134123

[3] Kroth PJ, Morioka-Douglas N, Veres S, . Association of electronic health record design and use factors with clinician stress and burnout. JAMA Netw Open. 2019;2(8):e199609. doi:10.1001/jamanetworkopen.2019.9609 31418810 PMC6704736

[4] Johnson KB, Neuss MJ, Detmer DE. Electronic health records and clinician burnout: a story of three eras. J Am Med Inform Assoc. 2021;28(5):967-973. doi:10.1093/jamia/ocaa274 33367815 PMC8068425

[5] Downing NL, Bates DW, Longhurst CA. Physician burnout in the electronic health record era: are we ignoring the real cause? Ann Intern Med. 2018;169(1):50-51. doi:10.7326/M18-0139 29801050

[6] Shultz CG, Holmstrom HL. The use of medical scribes in health care settings: a systematic review and future directions. J Am Board Fam Med. 2015;28(3):371-381. doi:10.3122/jabfm.2015.03.140224 25957370

[7] Rotenstein L, Melnick ER, Iannaccone C, . Virtual scribes and physician time spent on electronic health records. JAMA Netw Open. 2024;7(5):e2413140. doi:10.1001/jamanetworkopen.2024.13140 38787556 PMC11127114

[8] Owens LM, Wilda JJ, Grifka R, Westendorp J, Fletcher JJ. Effect of ambient voice technology, natural language processing, and artificial intelligence on the patient-physician relationship. Appl Clin Inform. 2024;15(4):660-667. doi:10.1055/a-2337-4739 38834180 PMC11305826

[9] Galloway JL, Munroe D, Vohra-Khullar PD, . Impact of an artificial intelligence-based solution on clinicians’ clinical documentation experience: initial findings using ambient listening technology. J Gen Intern Med. 2024;39(13):2625-2627. doi:10.1007/s11606-024-08924-2 38980463 PMC11436573

[10] Balloch J, Sridharan S, Oldham G, . Use of an ambient artificial intelligence tool to improve quality of clinical documentation. Future Healthc J. 2024;11(3):100157. doi:10.1016/j.fhj.2024.100157 39371531 PMC11452835

[11] Haberle T, Cleveland C, Snow GL, . The impact of nuance DAX ambient listening AI documentation: a cohort study. J Am Med Inform Assoc. 2024;31(4):975-979. doi:10.1093/jamia/ocae022 38345343 PMC10990544

[12] Liu TL, Hetherington TC, Dharod A, . Does AI-powered clinical documentation enhance clinician efficiency? A longitudinal study. NEJM AI. 2024. doi:10.1056/AIoa2400659

[13] Hyzy M, Bond R, Mulvenna M, . System usability scale benchmarking for digital health apps: meta-analysis. JMIR Mhealth Uhealth. 2022;10(8):e37290. doi:10.2196/37290 35980732 PMC9437782

[14] Adams C, Walpola R, Schembri AM, Harrison R. The ultimate question? Evaluating the use of net promoter score in healthcare: a systematic review. Health Expect. 2022;25(5):2328-2339. doi:10.1111/hex.13577 35985676 PMC9615049

[15] Apathy NC, Holmgren AJ, Cross DA. Physician EHR time and visit volume following adoption of team-based documentation support. JAMA Intern Med. 2024;184(10):1212-1221. doi:10.1001/jamainternmed.2024.4123 39186284 PMC11348094

[16] Rule A, Florig ST, Bedrick S, Mohan V, Gold JA, Hribar MR. Comparing scribed and non-scribed outpatient progress notes. AMIA Annu Symp Proc. 2022;2021:1059-1068. 35309010 PMC8861667

[17] TrendBurden: pulse survey on excessive documentation burden for health professionals. American Medical Informatics Association. Accessed November 24, 2024. https://amia.org/about-amia/amia-25x5/trendburden-pulse-survey

