# Supplementary material for: Clinician Experiences With Ambient Scribe Technology to Assist With Documentation Burden and Efficiency
Source: JAMA Netw Open. 2025 Feb 19;8(2):e2460637. doi: 10.1001/jamanetworkopen.2024.60637 (PMC11840636; doi:10.1001/jamanetworkopen.2024.60637)
Supplement: Supplement 1. — eFigure. Number of Clinicians Using Ambient Scribing Week to Week Over the Course of the Study eTable. Signal Data Analysis with Alternate Random-Effects Models eMethods [file jamanetwopen-e2460637-s001.pdf]

Supplementary Online Content

Duggan MJ, Gervase J, Schoenbaum A, et al. Clinician experiences with ambient scribe technology to assist with documentation burden and efficiency. *JAMA Netw Open*. 2025;8(2):e2460637. doi:10.1001/jamanetworkopen.2024.60637

eFigure. Number of Clinicians Using Ambient Scribing Week to Week Over the Course of the Study

eTable. Signal Data Analysis with Alternate Random-Effects Models

eMethods

This supplementary material has been provided by the authors to give readers additional information about their work.

© 2025 Duggan MJ et al. *JAMA Network Open*.

eFigure. Number of Clinicians Using Ambient Scribing Week to Week Over the Course of the Study

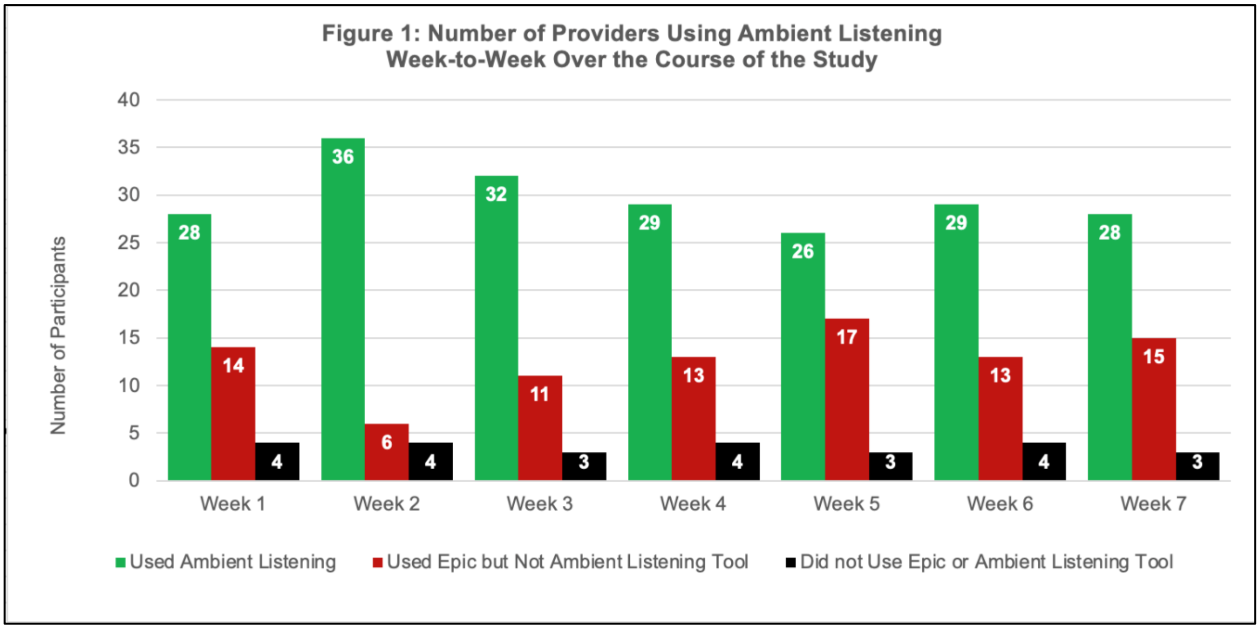

Green indicates clinicians who used DAX Copilot (Epic with ambient scribing) during a given week. Red indicates clinicians who engaged in outpatient charting in Epic but did not use ambient scribing during that week. Black indicates clinicians who were out of office, defined as zero outpatient charting in Epic and no ambient scribing use that week.

© 2025 Duggan MJ et al. *JAMA Network Open*.

eTable. Signal Data Analysis with Alternate Random-Effects Models

Primary Model: 39 week of baseline data, all covarites included, "No-DAX Usage "weeks in post period excluded								
**Metric**	**39-Week Baseline (Pre-DAX) Mean**	**Estimate (β)**	**Δ**	**Standard Error**	**95% Confidence Interval**	**P Value**	**Log-Likelihood Function**	**Covariates Included**
**Average Time in Notes per Appointment (minutes)**	10.31	-1.56	-15.16%	0.39	[-2.32, -0.80]	**<0.001**	-5057.94	individual clinician, clinician role, specialty, patient complexity, clinician workload, clinician experience
**Appointments Closed Same Day (%)**	66.21	6.04	9.12%	1.60	[2.90, 9.18]	**<0.001**	-7584.42	individual clinician, clinician role, specialty, patient complexity, clinician workload, clinician experience
**Average After Hours Work Time per Scheduled Day (Pajama Time) (minutes)**	50.61	-8.56	-16.91%	3.63	[-15.67, -1.45]	**0.02**	-8371.38	individual clinician, clinician role, specialty, patient complexity, clinician workload, clinician experience
**Portion of Notes Manually Typed (%)**	11.23	-2.33	-20.72%	0.42	[-3.14, -1.51]	**<0.001**	-5259.87	individual clinician, clinician role, specialty, patient complexity, clinician

© 2025 Duggan MJ et al. *JAMA Network Open*.

								workload, clinician experience
**Total Length of All Notes Generated by Provider (characters)**	202637.46	31389.95	15.49%	5379.29	[20846.73, 41933.17]	**<0.001**	-21643.88	individual clinician, clinician role, specialty, patient complexity, clinician workload, clinician experience

Alternate Model A: 39 week of baseline data, all covarites included, no exclusions of "No-DAX Usage" in the post-DAX period

Metric	39-Week Baseline (Pre-DAX) Mean	Estimate (β)	Δ	Standard Error	95% Confidence Interval	P Value	Log-Likelihood Function	Covariates Included
**Average Time in Notes per Appointment (minutes)**	10.31	-1.49	-14.43%	0.33	[-2.13, -0.85]	**<0.001**	-5252.23	individual clinician, clinician role, specialty, patient complexity, clinician workload, clinician experience
**Appointments Closed Same Day (%)**	66.21	2.99	4.52%	1.36	[0.33, 5.66]	**0.03**	-7904.71	individual clinician, clinician role, specialty, patient complexity, clinician workload, clinician experience
**Average After Hours Work Time per Schedule**	50.61	-4.63	-9.14%	3.05	[-10.61, 1.36]	0.13	-8685.83	individual clinician, clinician role, specialty, patient complexity, clinician workload,

© 2025 Duggan MJ et al. *JAMA Network Open*.

**d Day (Pajama Time) (minutes)**								clinician experience
**Portion of Notes Manually Typed (%)**	11.23	-2.25	-20.06%	0.35	[-2.94, -1.56]	**<0.001**	-5487.70	individual clinician, clinician role, specialty, patient complexity, clinician workload, clinician experience
**Total Length of All Notes Generated by Provider (characters)**	202637.46	17489.73	8.63%	4526.85	[8617.26, 26362.19]	**<0.001**	-22510.29	individual clinician, clinician role, specialty, patient complexity, clinician workload, clinician experience

Alternate Model B: 10 week of baseline data, all covarites included, "No-DAX Usage "weeks in post period excluded

Metric	10-Week Baseline (Pre-DAX) Mean	Estimate (β)	Δ	Standard Error	95% Confidence Interval	P Value	Log-Likelihood Function	Covariates Included
**Average Time in Notes per Appointment (minutes)**	9.67	-1.09	-11.22%	0.33	[-1.73, -0.44]	**0.001**	-1481.44	individual clinician, clinician role, specialty, patient complexity, clinician workload, clinician experience
**Appointments Closed Same Day (%)**	65.05	7.36	11.32%	1.86	[3.73, 11.00]	**<0.001**	-2434.71	individual clinician, clinician role, specialty, patient complexity,

© 2025 Duggan MJ et al. *JAMA Network Open*.

								clinician workload, clinician experience
**Average After Hours Work Time per Scheduled Day (Pajama Time) (minutes)**	46.65	-5.84	-12.52%	3.07	[-11.86, 0.18]	0.06	-2550.32	individual clinician, clinician role, specialty, patient complexity, clinician workload, clinician experience
**Portion of Notes Manually Typed (%)**	10.31	-1.69	-16.44%	0.39	[-2.47, -0.92]	**<0.001**	-1596.09	individual clinician, clinician role, specialty, patient complexity, clinician workload, clinician experience
**Total Length of All Notes Generated by Provider (characters)**	219380.65	17286.31	7.88%	6098.10	[5334.24, 29238.37]	**0.005**	-6812.19	individual clinician, clinician role, specialty, patient complexity, clinician workload, clinician experience

© 2025 Duggan MJ et al. *JAMA Network Open*.

eMethods

ChatGPT Prompt for Qualitative Analysis

The following prompt was used to for the ChatGPT-assisted identification of qualitative feedback themes: “You are a medical quality improvement researcher. You recently piloted an ambient scribing tool to help physicians with documenting outpatient appointments, and you are analyzing the physicians' feedback for publication in a medical journal. Summarize the key themes in of the qualitative feedback below, and discuss the physicians' overall impression of the ambient scribing tool DAX for publication:.” This statement was followed by all of the qualitative feedback obtained from the post-survey listed in the order that the post-surveys were completed by participating clinicians.

© 2025 Duggan MJ et al. *JAMA Network Open*.
