# Supplementary material for: Clinician Experiences With Ambient Scribe Technology to Assist With Documentation Burden and Efficiency
Source: JAMA Netw Open. 2025 Feb 19;8(2):e2460637. doi: 10.1001/jamanetworkopen.2024.60637 (PMC11840636; doi:10.1001/jamanetworkopen.2024.60637)
Supplement: Supplement 2. — Data Sharing Statement [file jamanetwopen-e2460637-s002.pdf]

Data Sharing Statement

Duggan. Clinician Experiences With Ambient Scribe Technology to Assist With Documentation Burden and Efficiency. *JAMA Netw Open*. Published February 19, 2025. doi:10.1001/jamanetworkopen.2024.60637

Data
Data available: No

Additional Information
Explanation for why data not available: The data used for this study are part of the Epic Signal dataset and are not unidentified at the provider or patient level.
